# Enhancement of Sweet Corn Seed Quality and Early Seedling Vigor by *Priestia* sp. RMT2NF4: Functional and Genomic Characterization of a Plant Growth-Promoting Strain

**DOI:** 10.3390/microorganisms14071388

**Published:** 2026-06-23

**Authors:** Tawanchai Khuendee, Yupa Chromkaew, Nuttapon Khongdee, Rattanaphon Chaima, Phanumat Ainta, Narin Iamthongin, Nichakarn Pota, Benyapa Kitwetch, Toungporn Uttarotai

**Affiliations:** 1 Department of Plant and Soil Science, Chiang Mai University, Chiang Mai 50200, Thailand; 2 Center of Excellence on Agricultural Biotechnology: (AG-BIO/MHESI), Bangkok 10900, Thailand; 3 Agrobiodiversity in Highland and Sustainable Utilization Research Group, Faculty of Agriculture, Chiang Mai University, Chiang Mai 50200, Thailand; 4 Department of Highland Agriculture and Natural Resources, Faculty of Agriculture, Chiang Mai University, Chiang Mai 50200, Thailand; 5 Department of Agricultural Innovation Research, Integration, Demonstration and Training Center, Chiang Mai University, Chiang Mai 50200, Thailand; 6 Multidisciplinary and Interdisciplinary School, Chiang Mai University, Chiang Mai 50200, Thailand

**Keywords:** *Priestia* sp., sweet corn, seed vigor, plant growth-promoting bacteria, whole-genome sequencing, bioinoculant

## Abstract

The development of sustainable microbial inoculants for crop production requires strains with demonstrated plant growth-promoting performance and well-characterized functional potential. This study evaluated the effect of *Priestia* sp. RMT2NF4, isolated from the rice rhizosphere, on sweet corn (*Zea mays* L.) seed physiological quality and early seedling vigor, supported by whole-genome sequencing analysis. Seed treatment effects were evaluated using a between-paper germination assay under controlled conditions at 25 °C for 7 days. Seed treatment with RMT2NF4 significantly increased germination percentage, germination index, and seedling growth rate by 13.26%, 21.30%, and 23.71%, respectively (*p* < 0.05). Inoculated seedlings also exhibited significantly greater shoot length, while root length and abnormal seedling proportion showed numerical but non-significant improvements. Genomic analysis identified genes putatively associated with tryptophan biosynthesis, nutrient acquisition, and stress adaptation. The integration of phenotypic validation and genome-informed functional profiling highlights the potential of RMT2NF4 and provides a basis for further evaluation of RMT2NF4 as a candidate plant growth-promoting bacterium to support sustainable sweet corn production and reduce reliance on chemical inputs.

## 1. Introduction

The rhizosphere represents a dynamic interface where plant roots interact closely with diverse microbial communities, forming a critical zone that regulates plant growth, development, and stress responses [1,2,3,4].

Microorganisms inhabiting this region are strongly influenced by root exudates and, in turn, modulate plant physiology through biochemical signaling and nutrient transformation processes [5]. These plant–microbe interactions are particularly important during early developmental stages, when seedlings exhibit high physiological plasticity and are highly responsive to microbe-derived signals such as phytohormones, nutrient mobilization products, and stress-related metabolites [6]. Consequently, rhizosphere microorganisms play a decisive role in root–shoot architecture formation, seedling establishment, and subsequent crop performance [7].

Climate variability, including rising temperatures and irregular precipitation patterns, has intensified abiotic stress during early plant growth stages [8]. Seed germination and seedling establishment are among the most vulnerable phases of the plant life cycle, where even moderate environmental stress can significantly reduce vigor and stand uniformity [9,10,11]. In conventional agricultural systems, these constraints are often mitigated through increased application of chemical fertilizers and pesticides to stabilize yields; however, such practices may disrupt soil microbial communities and contribute to environmental contamination and long-term soil degradation [12,13]. Biological regulation mediated by beneficial rhizosphere microorganisms has therefore gained increasing attention as a sustainable strategy to enhance early plant development under changing environmental conditions [7].

Plant growth-promoting rhizobacteria (PGPR) constitute a functionally diverse group of root-associated bacteria capable of influencing plant performance through both direct and indirect mechanisms, including phytohormone production, nutrient mobilization, and modulation of plant stress responses [6]. Several PGPR strains, such as *Azospirillum brasilense*, *Bacillus subtilis*, and *Pseudomonas fluorescens*, have been reported to improve seed germination, root elongation, nutrient uptake, and early seedling vigor in sweet corn and related crops [14,15]. Sweet corn was selected because rapid and uniform seedling establishment is essential for stand uniformity and subsequent crop productivity. The early germination stage is also highly responsive to seed-applied plant growth-promoting bacteria, making sweet corn a suitable crop system for evaluating the effect of RMT2NF4 on seed physiological quality and early seedling vigor. In this study, seed quality refers specifically to physiological seed quality associated with germination percentage, germination index, seedling growth rate, root and shoot development, and abnormal seedling proportion, rather than seed compositional or nutritional quality. However, plant growth responses to PGPR are often strain-specific, reflecting differences in metabolic capacity, colonization efficiency, and functional traits [16]. Linking observed plant phenotypes to strain-level functional potential therefore remains a key challenge in translating PGPR research into reliable agricultural applications. Although several PGPR have been evaluated in maize and sweet corn, strain-level information on *Priestia* spp. in these crop systems remains limited. In particular, the relationship between *Priestia*-mediated seed treatment, early seedling vigor, and genome-inferred plant-associated traits is still insufficiently characterized.

The genus *Priestia*, recently reclassified from the *Bacillus* clade based on phylogenomic and phenotypic evidence [17], has emerged as a promising yet comparatively underexplored group of rhizosphere-associated bacteria. Species within this genus have been isolated from agricultural soils, plant rhizospheres, and contaminated environments [18,19], and several strains have demonstrated plant-beneficial properties, including indole-3-acetic acid (IAA) production, mineral nutrient solubilization, nitrogen metabolism, and tolerance to abiotic stress [6,20]. Despite these reported capabilities, the functional basis and strain-specific mechanisms by which *Priestia* species influence early plant development remain insufficiently characterized, particularly in economically important crops [21].

*Priestia* sp. strain RMT2NF4 was originally isolated from the rice rhizosphere and was previously characterized as a plant growth-promoting bacterium [22]. In the previous study, preliminary identification was performed based on colony and cell morphology together with 16S rRNA gene sequence analysis, and the strain was reported to exhibit plant growth-promoting traits, including indole-3-acetic acid (IAA) production, phosphate solubilization, and nitrogenase activity. However, its effect on sweet corn seed’s physiological quality and early seedling vigor, together with whole-genome-based taxonomic placement and functional characterization, had not been previously reported. Therefore, the novelty of the present study lies in integrating controlled seed-treatment assays with whole-genome-based analysis to assess the early growth-promoting potential of RMT2NF4 in sweet corn. We hypothesized that seed treatment with RMT2NF4 would improve sweet corn seed physiological quality and early seedling vigor and that whole-genome analysis would reveal putative plant-associated traits potentially linked to these observed responses. Therefore, the objectives of this study were to evaluate the effects of *Priestia* sp. RMT2NF4 on sweet corn (*Zea mays* L. *saccharata*) seed quality and early seedling development and to employ whole-genome sequencing and functional annotation to associate observed phenotypic responses with the strain’s genomic potential, thereby advancing strain-level understanding of *Priestia*-mediated growth promotion in agricultural systems.

## 2. Materials and Methods

### 2.1. Strain and Culture Conditions

*Priestia* sp. strain RMT2NF4 was originally isolated from the rice rhizosphere in Mae Taeng District, Chiang Mai Province, northern Thailand. For plant growth-promoting evaluation, the strain RMT2NF4 was cultured in nutrient broth (NB; HiMedia Laboratories Pvt. Ltd., PA, USA) at 30 °C with shaking at 121 rpm (New Brunswick Innova 2300, NJ, USA) for 72 h following the previously established culture conditions for this strain [22]. The shaking speed was used to maintain aeration and suspension homogeneity during bacterial growth. Optical density at 600 nm (OD600) was not used for inoculum standardization in this study. Instead, bacterial population density was quantified using a serial dilution drop-plate method [23]. Briefly, the bacterial suspension was serially diluted in sterile distilled water, and 10 µL aliquots from appropriate dilutions were dropped onto nutrient agar plates. Plates were incubated at 28–30 °C for 48–72 h, and visible colonies were counted to estimate the bacterial population. The final inoculum density was adjusted to approximately 1 × 10^7^ CFU mL^−1^ before seed treatment. The resulting bacterial suspension was used for seed inoculation assays. For laboratory working stock maintenance, the strain was stored at −20 °C in NB supplemented with glycerol as a cryoprotectant. This storage condition was used for routine laboratory maintenance rather than formal long-term repository preservation [24].

### 2.2. Molecular Identification by 16S rRNA Gene Sequencing

Genomic DNA was extracted from RMT2NF4 cells grown on Nutrient Agar (NA) using the FavorPrep Tissue Genomic DNA Extraction Mini Kit (Favorgen, Taipei, Taiwan) following the manufacturer’s protocol. The nearly full-length 16S rRNA gene was amplified by polymerase chain reaction (PCR) using universal primers 27F (5′-AGAGTTTGATCMTGGCTCAG-3′) and 1525R (5′-AAGGAGGTGWTCCARCC-3′) [25].

PCR amplification was performed in a GeneAmp PCR System 9700 (Applied Biosystems, CA, USA) under the following conditions: initial denaturation at 94 °C for 2 min; 30 cycles of denaturation at 94 °C for 30 s, annealing at 55 °C for 30 s, and extension at 72 °C for 30 s; followed by a final extension at 72 °C for 7 min. Amplified products were verified by electrophoresis on a 1% (*w*/*v*) agarose gel. Purified PCR products were subjected to Sanger sequencing at BTSeq DNA Sequencing (Seoul, Republic of Korea). The resulting 16S rRNA gene sequence was compared against the NCBI database using BLAST version 2.15.0 to determine the taxonomic affiliation of strain RMT2NF4. The nearly full-length 16S rRNA gene sequence of strain RMT2NF4 (1496 bp) was successfully amplified and sequenced. The accession number was verified in the GenBank database, and the resulting sequence is publicly available under accession number PX471528.

### 2.3. Seed Germination Assay

Seed germination performance was evaluated using the standard between-paper (BP) method following the International Seed Testing Association guidelines [26]. A total of 2000 sweet corn seeds of the commercial hybrid cultivar “Hi-Brix 59” (commercial hybrid variety; Pacific Seeds Company, Saraburi, Thailand) were equally divided into two treatments: (i) seeds soaked in a suspension of *Priestia* sp. RMT2NF4 prepared as described in Section 2.1 and (ii) control seeds soaked in sterile distilled water. Seeds were immersed for 30 min following a protocol adapted from Moussa [27].

For each treatment, 20 independent experimental replicates were prepared, with each replicate consisting of 50 seeds. Seeds were randomly assigned to the control and RMT2NF4 treatments before soaking. After treatment, seeds were evenly placed on germination paper sheets (54 × 30 cm) moistened with 30 mL of sterile distilled water. The papers were rolled, sealed in plastic bags to minimize moisture loss, and incubated at 25 °C for 7 days under controlled laboratory germination conditions. Relative humidity was not actively controlled; however, moisture availability was maintained by sealing the rolled germination papers in plastic bags. Germination index (GI) was assessed on days 4 and 7, whereas germination percentage (GP), seedling growth rate (SGR), shoot length, root length, and abnormal seedling proportion were determined on day 7. Root and shoot lengths were measured using normal seedlings only, as defined by International Seed Testing Association (ISTA) criteria.

### 2.4. Seedling Quality Assessment

Seedling quality was evaluated based on germination index (GI), germination percentage (GP), seedling growth rate (SGR), and the proportion of abnormal seedlings. The germination index (GI) was calculated according to the Association of Official Seed Analysts [28] using the following equation:

GI = ∑n/t

where n represents the number of normal seedlings recorded on a given day (day 4 or day 7), and t represents the corresponding day after sowing.

Germination percentage (GP) was calculated as the proportion of normal seedlings relative to the total number of seeds sown and expressed as a percentage [29]:

GP = (number of normal seedlings/total number of seeds sown) × 100

Seedling growth rate (SGR) was determined on day 7 as the total dry weight of normal seedlings divided by the number of normal seedlings and expressed as mg plant^−1^. Normal seedlings from each replicate were oven-dried until constant weight was achieved. Dry weight was measured using an analytical balance with a precision of 0.001 g. SGR was calculated using the following equation:

SGR = total dry weight of normal seedlings/number of normal seedlings

Abnormal seedlings were identified according to International Seed Testing Association (ISTA) criteria, and their proportion was calculated relative to the total number of germinated seedlings.

### 2.5. Whole-Genome Sequencing

Genomic DNA of *Priestia* sp. strain RMT2NF4 was extracted using a GF-1 Bacterial DNA Extraction Kit (Vivantis, Shah Alam, Malaysia). Paired-end libraries were prepared using the QIASEQ FX DNA Library Preparation Kit (Qiagen, MD, USA). Sequencing was performed at the Omics Science and Bioinformatics Center, Faculty of Science, Chulalongkorn University, Bangkok, Thailand, on an Illumina MiSeq platform, generating 2 × 250 bp paired-end reads.

Raw read quality was assessed using FastQC version 0.11.9 [30]. Adapter sequences and low-quality reads were removed using fastp version 0.23.2 [31]. The filtered reads were assembled de novo using Unicycler, which incorporates SPAdes for short-read assembly [32]. Assembly quality was evaluated based on genome size, number of contigs, N50, L50, GC content, and sequencing depth. Genome completeness and contamination were assessed using CheckM2 (v1.2.3) [33]. Plasmid status was obtained from the PATRIC/BV-BRC Comprehensive Genome Analysis assembly report generated from the SPAdes-based assembly workflow, which reported zero plasmids in the draft genome assembly. The assembled genome was deposited in the NCBI GenBank database under BioProject accession number PRJNA1219766.

### 2.6. Functional Annotation and Phylogenetic Analysis

Functional annotation and comparative genomic analyses were performed using the BV-BRC platform (version 3.53.3). Genome annotation was conducted using the RAST tool kit (RASTtk) pipeline [34], which applies a standardized gene-calling and functional classification framework across publicly available genomes. BLAST-based searches were conducted against curated specialty gene databases integrated within BV-BRC to identify genes putatively associated with plant growth-promoting traits, stress response, and environmental adaptation [35]. For comparative analysis of plant growth-promoting trait-related proteins, comparator genomes were selected based on their phylogenetic relatedness to strain RMT2NF4 and the availability of annotated genomes within the genus *Priestia*. Protein similarity comparisons were performed using BLASTP against selected reference genomes. The percentage identity values represent the best BLASTP matches for selected plant growth-promoting trait-related proteins. No fixed identity threshold was used to infer confirmed functional equivalence; therefore, the results were interpreted only as comparative protein similarity patterns rather than direct evidence of functional activity.

Taxonomic placement of strain RMT2NF4 was determined using whole-genome phylogenetic analysis. The closest reference and representative genomes were identified using Mash/MinHash distance estimation [36]. A total of 11 genomes, including strain RMT2NF4 and closely related representative *Priestia* genomes, were included in the phylogenomic analysis. The accession numbers of all genomes used for comparison are provided in Supplementary Table S2. Phylogenetic reconstruction was performed based on PATRIC global protein families (PGFams), aligned using MUSCLE [37]. Corresponding nucleotide sequences were mapped to the protein alignments, concatenated, and analyzed using RAxML with rapid bootstrapping to infer phylogenetic relationships and assess statistical support [38]. The analysis was performed through the standardized BV-BRC phylogenomic workflow; therefore, model parameters followed the platform default settings, and no user-specified substitution model was manually selected. Bootstrap support values were displayed on the phylogenomic tree where applicable.

Average nucleotide identity (ANI) values between strain RMT2NF4 and closely related reference genomes were calculated using the Orthologous Average Nucleotide Identity Tool (OAT) version 0.93.1 [39]. ANI values of approximately 95–96% were used as the commonly accepted threshold range for species-level relatedness.

### 2.7. Statistical Analysis

Data are presented as mean ± standard deviation (SD). Prior to ANOVA, the assumptions of normality and homogeneity of variance were evaluated using the Shapiro–Wilk test and Levene’s test, respectively. All parameters satisfied the assumptions required for ANOVA, and no data transformation was applied. The effect of seed treatment on individual germination and seedling quality parameters was analyzed using one-way analysis of variance (ANOVA), with seed treatment as the fixed factor. Because only two treatment groups were included, pairwise comparison was performed between the uninoculated control and the RMT2NF4-inoculated treatment. Exact *p*-values were reported for each parameter. Statistical significance was determined at *p* < 0.05. All statistical analyses were performed using Statistix software, version 10.0.

## 3. Results

### 3.1. Molecular Identification and Morphological Characterization

BLAST analysis of the amplified near full-length 16S rRNA gene sequence against the NCBI database revealed 100% sequence identity with several members of the genus *Priestia*, including *Priestia aryabhattai* strain B8W22. This 16S rRNA gene result was used as a preliminary taxonomic identification step. However, because the 16S rRNA gene is highly conserved within the *Priestia*/*Bacillus* clade, it does not provide sufficient resolution for definitive species-level assignment. Therefore, whole-genome phylogenetic analysis and ANI comparison were used as the primary taxonomic approaches in this study. Based on this conservative interpretation, the strain was retained as *Priestia* sp. RMT2NF4.

**Figure 1:**
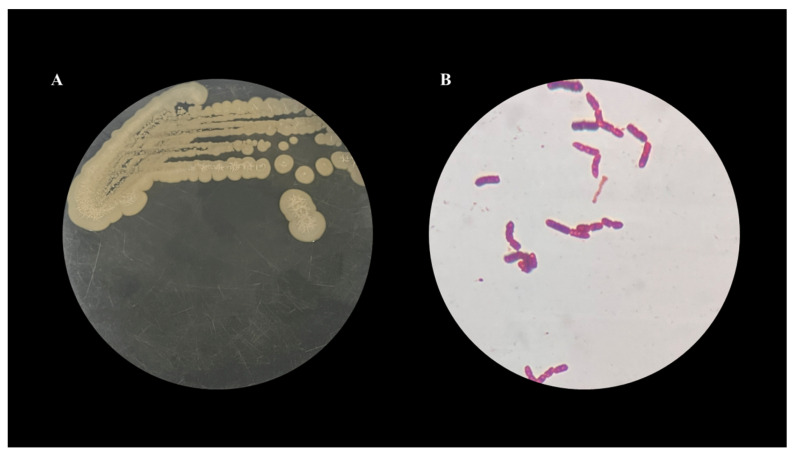
Morphological characteristics of *Priestia* sp. strain RMT2NF4: (**A**) Colony morphology on nutrient agar showing circular, smooth, creamy-white colonies. (**B**) Gram-stained cells displaying Gram-positive, rod-shaped morphology occurring predominantly as single cells or short chains.

Colony morphology on nutrient agar exhibited circular, smooth, creamy-white colonies. Microscopic examination of Gram-stained cells revealed Gram-positive, rod-shaped bacteria occurring predominantly as single cells or short chains (Figure 1). These phenotypic characteristics are consistent with members of the genus *Priestia*.

### 3.2. Effects of RMT2NF4 on Seed Germination and Early Seedling Vigor

Seed inoculation with *Priestia* sp. RMT2NF4 resulted in significant improvements in sweet corn germination performance compared with the untreated control (Table 1). Germination percentage (GP) increased from 74% in the control to 84% following RMT2NF4 treatment, corresponding to a 13.26% enhancement (*p* < 0.05).

The germination index (GI), which reflects both the rate and uniformity of seed germination, increased by 21.30% in inoculated seeds relative to the control (*p* < 0.05). In addition, seedling growth rate (SGR), calculated based on seedling dry biomass accumulation, showed a 23.71% increase under bacterial treatment (*p* < 0.05), indicating improved early biomass development (Table 1).

Evaluation of seedling morphological parameters further supported these findings (Table 1). Shoot length was significantly greater in RMT2NF4-treated seedlings compared with the control (*p* < 0.05). Root length increased numerically from 10.38 ± 2.00 cm in the control to 12.38 ± 2.36 cm in the RMT2NF4 treatment, corresponding to a 19.33% increase; however, this difference was not statistically significant (*p* = 0.16). The proportion of abnormal seedlings was numerically lower in the RMT2NF4 treatment group than in the control, decreasing from 21.8 ± 8.58% to 11.6 ± 7.21%; however, this difference was not statistically significant (*p* = 0.2282). Therefore, the lower abnormal seedling proportion should be interpreted as a non-significant trend rather than a confirmed treatment effect.

**Figure 2:**
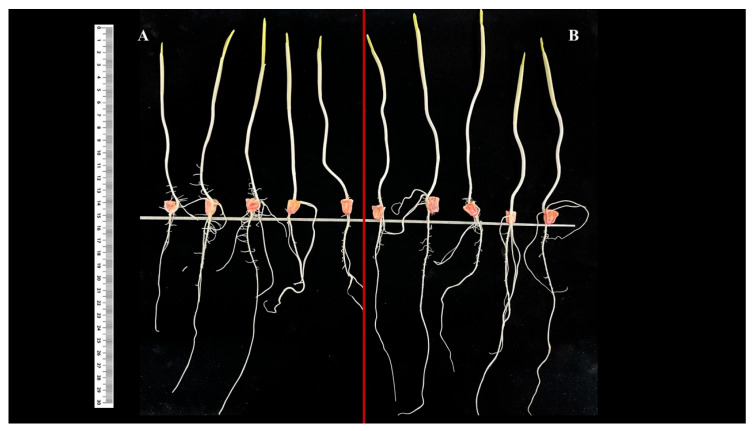
Representative sweet corn seedlings from the uninoculated control and RMT2NF4-treated groups after 7 days of incubation under the between-paper germination assay. (**A**) Control group; (**B**) RMT2NF4-treated group. Quantitative seedling growth parameters, including shoot length, root length, germination percentage, germination index, and seedling growth rate, are presented in Table 1. Ruler unit: cm.

Visual assessment on day 7 corroborated the quantitative measurements (Figure 2). Seedlings derived from RMT2NF4-treated seeds displayed more uniform development and greater overall vigor compared with untreated controls.

**Figure 3:**
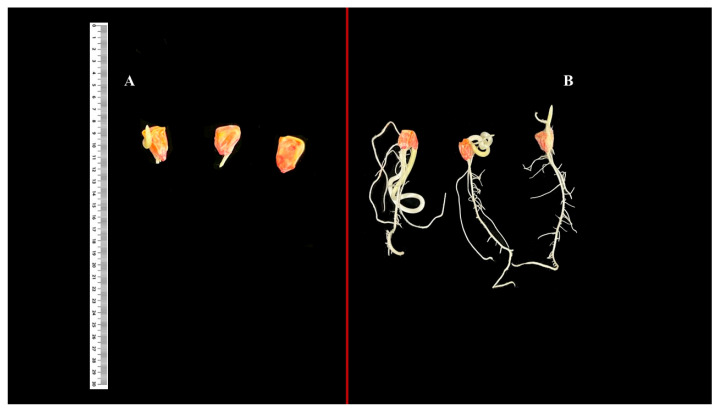
Representative normal and abnormal sweet corn seedlings observed after 7 days of incubation. (**A**) Control group; (**B**) RMT2NF4-treated group. Abnormal seedlings were classified according to ISTA criteria. The quantitative proportion of abnormal seedlings is presented in Table 1. Ruler unit: cm.

Representative abnormal phenotypes observed in both treatments are shown in Figure 3. In the control group, abnormal seedlings frequently exhibited severely reduced root and shoot development, whereas abnormal seedlings from the RMT2NF4 treatment group commonly showed radicle development with limited plumule elongation. Collectively, these results indicate that RMT2NF4 treatment significantly improved several early seed physiological and seedling vigor parameters in sweet corn, particularly germination percentage, germination index, seedling growth rate, and shoot length, while root length and abnormal seedling proportion showed numerical but non-significant improvements.

### 3.3. Genome Assembly and Taxonomic Placement of Strain RMT2NF4

**Figure 4:**
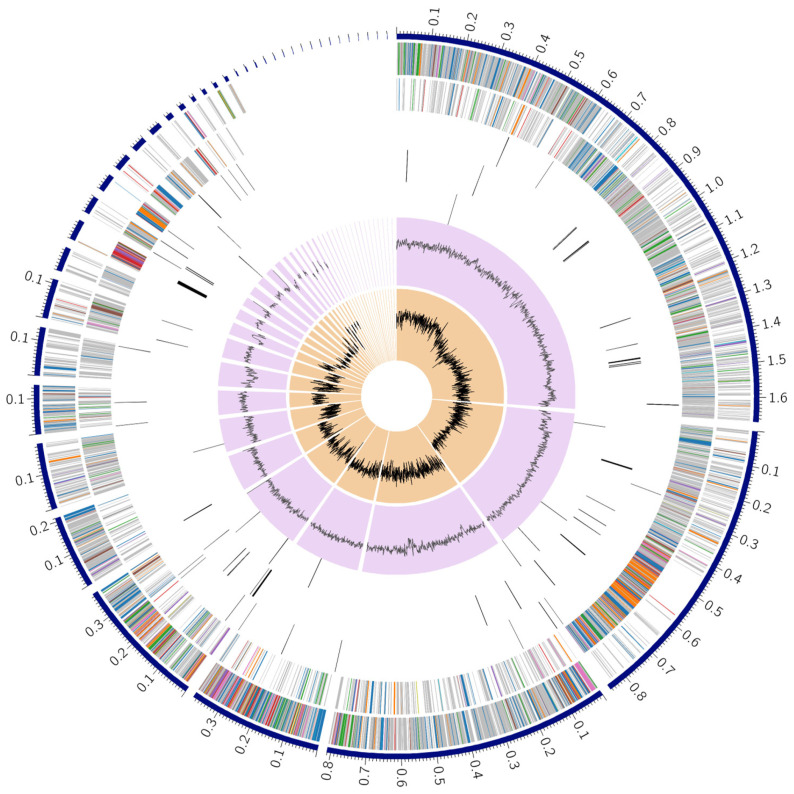
Circular genome map of *Priestia* sp. strain RMT2NF4 based on de novo assembly.

The draft genome of strain RMT2NF4 consisted of 40 contigs, with a total genome size of 5,318,699 bp and a GC content of 37.89% (Figure 4). The assembly achieved an estimated sequencing coverage of 30× and had an N50 value of 811,292 bp and an L50 value of 3, indicating moderate contiguity for a short-read draft genome. Genome annotation identified 5698 coding sequences, 4 rRNA genes, and 68 tRNA genes. The relatively low number of detected rRNA genes may reflect the fragmented nature of the draft assembly and the difficulty of resolving repetitive rRNA operons using short-read sequencing. Genome quality assessment indicated an estimated completeness of 99.27% and contamination of 2.96%, supporting the overall quality of the assembled draft genome (Table 2). 

**Figure 5:**
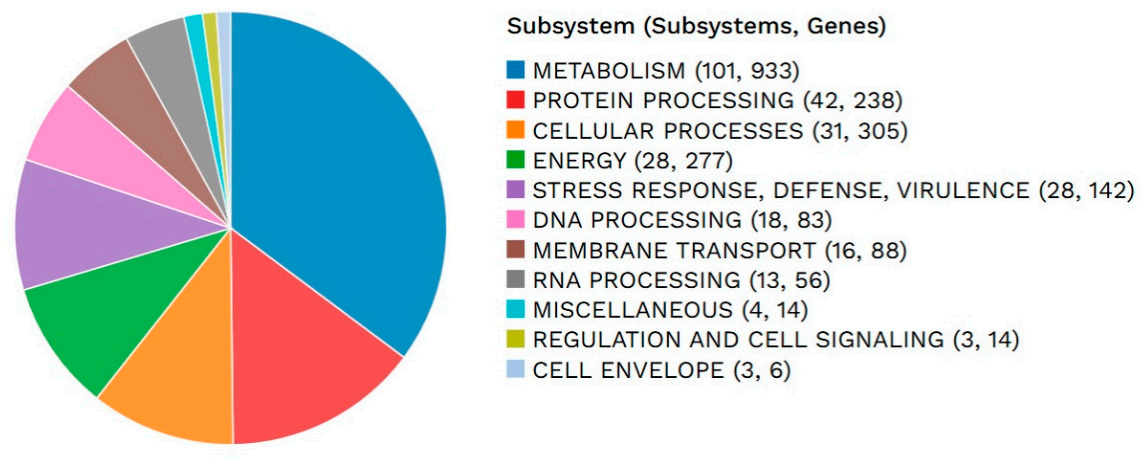
Distribution of annotated functional subsystem categories in the draft genome of *Priestia* sp. RMT2NF4 based on BV-BRC/RASTtk annotation. The pie chart represents the relative proportion of genes assigned to each functional subsystem category according to the numerical subsystem output generated by BV-BRC/RASTtk.

Subsystem-based functional classification using the BV-BRC annotation platform identified 11 major functional categories (Figure 5). The largest proportion of annotated genes was associated with Metabolism (933 genes), followed by Cellular Processes (305 genes), Energy (277 genes), and Protein Processing (238 genes). Additional functional groups included Stress Response, Defense, and Virulence (142 genes); Membrane Transport (88 genes); DNA Processing (83 genes); RNA Processing (56 genes); Regulation and Cell Signaling (14 genes); Miscellaneous Functions (14 genes); and Cell Envelope (6 genes).

**Figure 6:**
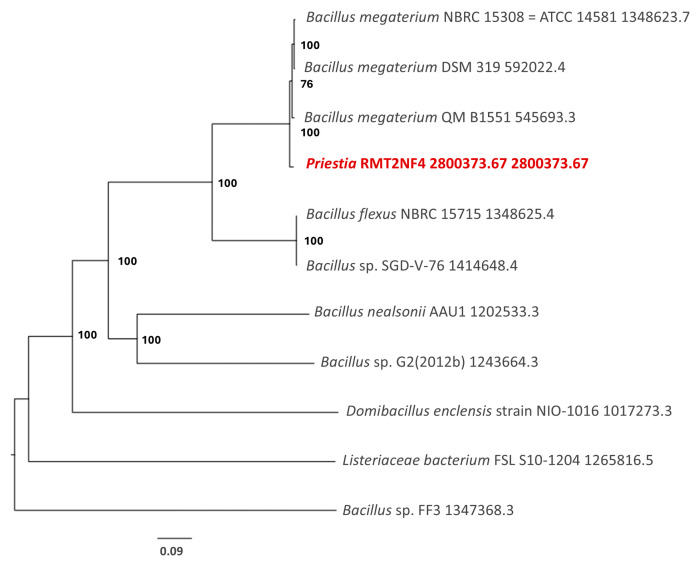
Phylogenomic tree based on whole-genome conserved protein families showing the taxonomic position of strain RMT2NF4 within the genus *Priestia*. Strain RMT2NF4 is highlighted in red.

Phylogenomic analysis based on whole-genome conserved protein families positioned strain RMT2NF4 within the genus *Priestia* (Figure 6). The strain clustered most closely with reference genomes assigned to *Priestia megaterium* (synonym *Bacillus megaterium*), indicating close evolutionary relatedness.

**Figure 7:**
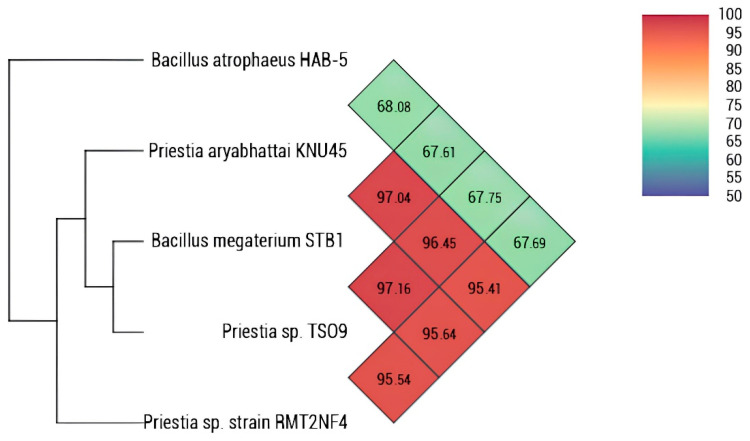
Heatmap of average nucleotide identity (ANI) values between strain RMT2NF4 and closely related *Priestia* and *Bacillus* reference genomes, calculated using the Orthologous Average Nucleotide Identity Tool (OAT).

To further evaluate species-level affiliation, average nucleotide identity (ANI) analysis was performed using the Orthologous Average Nucleotide Identity Tool (OAT). The resulting heatmap (Figure 7) showed that strain RMT2NF4 shared the highest ANI value (95.64%). Although strain RMT2NF4 showed the highest ANI value with *Priestia megaterium* STB1, the value of 95.64% falls near the commonly used species boundary. Because digital DNA–DNA hybridization was not performed in this study, we retained the conservative designation *Priestia* sp. RMT2NF4 rather than formally assigning the strain to the *P. megaterium* lineage. The strain is therefore described as closely related to the *P*. *megaterium* lineage. This value falls within the generally accepted species boundary range (95–96%), supporting its placement within the *P. megaterium* lineage while indicating modest genomic divergence from the reference strain.

### 3.4. Functional Genomic Traits Associated with Plant Growth Promotion

#### 3.4.1. Tryptophan Biosynthesis and IAA-Related Potential

Genome analysis of strain RMT2NF4 revealed the presence of a complete tryptophan biosynthesis gene cluster, including *trpA*, *trpB*, *trpC*, *trpD*, and *trpE* (Supplementary Table S1). These genes encode enzymes responsible for the stepwise conversion of chorismate to L-tryptophan, a central precursor for indole-3-acetic acid (IAA) biosynthesis in many plant-associated bacteria.

Comparative genomic analysis demonstrated high sequence similarity (96.5–99.7%) of the *trp* gene cluster among closely related *Priestia* species, indicating conservation of this pathway within the genus. In contrast, lower similarity was observed when compared with selected *Bacillus* strains, suggesting phylogenetic divergence outside the *Priestia* lineage.

In addition to the core tryptophan biosynthetic genes, the genome encodes a predicted auxin efflux carrier protein belonging to the auxin efflux carrier family (Supplementary Table S1). The presence of this predicted transporter suggests a possible auxin-related transport potential; however, auxin export activity was not experimentally validated in this study.

**Figure 8:**
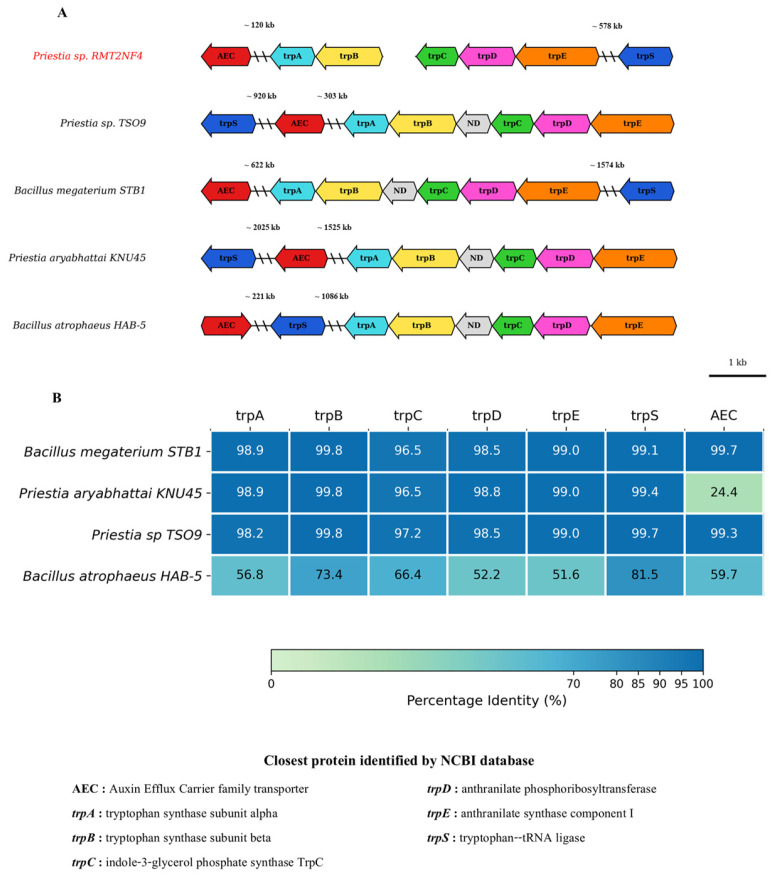
Comparative protein similarity of selected plant growth-promoting trait-related proteins in *Priestia* sp. RMT2NF4 and representative *Priestia* genomes. (**A**) Comparator strains were selected based on phylogenetic relatedness and genome availability. (**B**) Percentage values indicate BLASTP amino acid identity of the best-matching proteins and should be interpreted as comparative similarity rather than direct evidence of functional activity.

The genomic organization of the *trp* gene cluster in strain RMT2NF4 and its comparison with selected plant growth-promoting bacteria are shown in Figure 8. The conserved arrangement of these genes supports the functional integrity of the pathway.

Although the genome does not independently confirm active IAA production, the presence of a complete tryptophan biosynthetic pathway and auxin-associated transport machinery provides a genomic framework consistent with the previously reported IAA-producing phenotype of strain RMT2NF4. This genetic potential is consistent with the observed improvement in germination index and seedling growth rate following seed inoculation; however, a direct causal relationship between these genome-inferred traits and the observed seedling responses was not experimentally demonstrated in this study. 

#### 3.4.2. Nutrient Acquisition Systems

Genome analysis of strain RMT2NF4 identified several genes associated with nitrogen metabolism and phosphate acquisition (Supplementary Table S1), indicating potential adaptation to nutrient-limited rhizosphere environments.

Genes belonging to the fix gene family, including *fixA*, *fixB*, and *fixH*, were detected in the genome. These genes are commonly associated with electron transfer processes linked to nitrogen metabolism. In addition, a NifU family protein was identified, which is involved in the assembly of iron–sulfur (Fe–S) clusters required for the function of various metabolic enzymes. Although the complete nitrogenase structural gene set (e.g., *nifHDK*) was not detected, the presence of nitrogen metabolism-related genes suggests a potential role in nitrogen cycling processes rather than direct atmospheric nitrogen fixation.

The genome also harbors a complete high-affinity phosphate transport system (*pst* operon), including *pstA*, *pstB*, *pstC*, and *pstS* genes, as well as regulatory components such as *phoU*. These genes are typically involved in phosphate uptake under low-phosphate conditions and may be relevant to phosphorus acquisition under soil or rhizosphere conditions, but this does not directly explain the paper-based germination response observed in this study.

Together, the presence of nitrogen metabolism-associated genes and phosphate transport systems indicates that strain RMT2NF4 possesses genome-inferred features potentially relevant to nutrient acquisition under rhizosphere conditions. However, because the present assay was conducted using a paper-based germination system, these genes should be interpreted as functional potential for future soil- or rhizosphere-based studies rather than as direct evidence explaining the observed seedling growth response.

#### 3.4.3. Motility and Root Colonization-Associated Traits

Genome analysis revealed a comprehensive set of genes associated with bacterial motility, chemotaxis, and surface attachment (Supplementary Table S1). Multiple gene clusters involved in flagellar biosynthesis were identified, including components of the *flh*, *fli*, and *flg* operons, which are essential for flagellar assembly and locomotion.

Genes associated with chemotactic signaling pathways, such as *che* family genes, were also detected, suggesting the capacity for directional movement toward root-derived exudates. Motility and chemotaxis are critical determinants of successful rhizosphere colonization, enabling bacteria to actively respond to chemical gradients in the root zone.

In addition, genes implicated in membrane transport and surface-associated processes were identified, which may contribute to bacterial adhesion and persistence in the rhizosphere environment. These genomic features collectively indicate that strain RMT2NF4 possesses the molecular machinery necessary for active movement and potential root association.

The presence of motility- and chemotaxis-related genes suggests colonization-related genomic potential. However, effective root colonization was not tested experimentally in this study; therefore, these genes should be interpreted as putative traits that may support plant association under appropriate conditions rather than as evidence of confirmed colonization ability.

#### 3.4.4. Stress Response, Osmotic Regulation, and Hormone-Related Genes

Genome analysis of strain RMT2NF4 identified a diverse set of genes associated with environmental stress tolerance and osmotic homeostasis (Supplementary Table S1). These include genes implicated in cold and heat stress response, osmoprotectant biosynthesis, ion transport, and potassium uptake systems. Such genes are commonly associated with bacterial survival under fluctuating temperature, water availability, and osmotic conditions typical of agricultural soils.

Notably, gene clusters related to osmotic balance, including potassium transport systems and compatible solute-associated pathways, were detected. These features may be associated with bacterial stress adaptation, but stress tolerance was not directly evaluated in this study.

The genome also contains genes encoding tRNA isopentenyltransferase-related proteins (miaA and miaB), which are involved in tRNA modification pathways linked to isoprenoid metabolism. While these genes are not direct evidence of cytokinin secretion, their presence suggests potential involvement in metabolic pathways associated with isoprenoid-derived compounds. Further functional validation would be required to clarify their role in plant hormone-related interactions.

Collectively, the presence of stress adaptation genes and hormone-related metabolic components indicates that strain RMT2NF4 possesses genome-inferred attributes potentially relevant to survival and functional persistence in the rhizosphere. However, these traits were not experimentally validated in this study and should not be interpreted as direct causes of the improved germination performance or early seedling vigor observed in inoculated sweet corn.

## 4. Discussion

The present study demonstrates that seed treatment with *Priestia* sp. strain RMT2NF4 improved early germination performance and seedling vigor in sweet corn under controlled conditions. Inoculation with RMT2NF4 significantly enhanced germination index (GI), seedling growth rate (SGR), shoot length, and germination percentage, while reducing the proportion of abnormal seedlings. Root length also increased numerically, although this difference was not statistically significant. These results suggest that RMT2NF4 mainly promoted early seedling vigor and seedling establishment rather than uniformly enhancing all growth parameters. Similar responses have been reported in maize–PGPR systems, where bacterial inoculation improved seedling vigor, biomass accumulation, and early growth performance more strongly than final germination percentage [40,41,42]. Such effects may reflect changes in early seedling growth dynamics during imbibition, radicle emergence, and seedling development [43,44].

The growth-promoting effect of RMT2NF4 is consistent with its previously reported plant growth-promoting traits, including IAA production, phosphate solubilization, and nitrogenase activity [22]. Bacterial IAA can influence root and shoot development in a concentration-dependent manner, with beneficial or inhibitory effects depending on the balance between microbial auxin production and host plant hormonal sensitivity [45,46,47,48]. However, IAA concentration was not quantified under the seed-treatment conditions used in the present study. Therefore, the observed seedling responses should be interpreted as potentially associated with previously reported IAA production rather than as direct evidence of auxin-mediated causation.

Whole-genome sequencing and comparative analyses placed strain RMT2NF4 within the genus *Priestia*. The ANI value of 95.64% with *P. megaterium* STB1 indicates close relatedness to the *P. megaterium* lineage; however, because this value is near the commonly used species boundary and digital DNA–DNA hybridization was not performed, the strain was conservatively retained as *Priestia* sp. RMT2NF4. The general genomic features of RMT2NF4 are consistent with previously reported *Priestia* genomes [49,50,51]. Genome annotation identified a complete *trp* gene cluster involved in tryptophan biosynthesis, which is relevant because tryptophan is a major precursor for bacterial IAA production [52]. This genomic feature is consistent with the previously reported IAA-producing phenotype of RMT2NF4 [22]. However, canonical marker genes for the five classical tryptophan-dependent IAA biosynthesis pathways were not clearly detected [53]. Therefore, the possibility of alternative or less-characterized IAA biosynthesis routes remains speculative and requires transcriptomic, metabolomic, or biochemical validation.

Several genome-inferred traits may be relevant to plant association, but they should not be interpreted as direct evidence of functional activity. The detection of a putative auxin efflux carrier suggests possible auxin-related transport potential, but auxin export was not experimentally confirmed. Similarly, genes associated with nitrogen metabolism, including *nifU* and *fixABH*, may indicate involvement in redox or nitrogen-related processes, but the absence of a complete nitrogenase structural gene set does not support the interpretation of RMT2NF4 as a primary nitrogen-fixing bacterium. The presence of phosphate transport and regulatory genes, including *pstABCS* and *phoHU*, suggests potential adaptation to phosphorus-limited environments [54,55]. However, because the present germination assay was conducted using the between-paper method without soil, nutrients available to seedlings were derived primarily from internal seed reserves. Therefore, nutrient acquisition-related genes are unlikely to directly explain the improved seedling growth observed under the present assay conditions and may be more relevant to future soil or rhizosphere performance.

The genome of RMT2NF4 also contained genes associated with motility, chemotaxis, and environmental stress adaptation. Flagellar assembly and chemotaxis genes may support bacterial movement toward root exudates and initial root surface contact under appropriate rhizosphere conditions [56]; however, these functions were inferred from genome annotation and were not experimentally confirmed in the present study. However, gene presence alone does not demonstrate effective root colonization, biofilm formation, or persistence on sweet corn roots. These functions require direct validation through root colonization assays, microscopy, or marker-based tracking. Likewise, genes related to thermal stress response, potassium transport, osmoprotectant biosynthesis, and ion homeostasis suggest potential stress-adaptation capacity, but stress tolerance was not directly evaluated in the present study. These genomic traits should therefore be interpreted as inferred functional potential rather than confirmed physiological performance.

Several plant-associated *Priestia* strains have been reported to promote plant growth through traits such as IAA production, phosphate solubilization, stress adaptation, and improved seedling development [49,50,51,57,58]. Strain RMT2NF4 shows a comparable plant-beneficial profile, as it previously exhibited IAA production, phosphate solubilization, and nitrogenase activity, while the present study demonstrates improved sweet corn seed physiological quality and early seedling vigor. Nevertheless, because no side-by-side comparison with other *Priestia* strains or commercial PGPR inoculants was conducted, RMT2NF4 cannot be concluded to be superior to previously reported strains. Instead, it should be considered a promising candidate for further validation.

Overall, this study integrates controlled seed-treatment assays with genome-informed analysis to characterize the early growth-promoting potential of *Priestia* sp. RMT2NF4 in sweet corn. The phenotypic results demonstrate improved seed physiological quality and early seedling vigor under controlled conditions, while genome analysis suggests potential plant-associated traits related to tryptophan biosynthesis, nutrient acquisition, motility, and stress adaptation. However, these genomic predictions remain inferential. Further studies involving quantitative IAA measurement, phosphate solubilization and uptake assays, motility and colonization assays, stress tolerance testing, transcriptomic or metabolomic analysis, and greenhouse or field validation are required to confirm the mechanisms underlying the observed seedling responses and to evaluate the practical bioinoculant potential of RMT2NF4.

## 5. Conclusions

This study demonstrates that seed treatment with *Priestia* sp. RMT2NF4 improved sweet corn seed physiological quality and early seedling vigor under controlled germination conditions. RMT2NF4 treatment increased germination percentage, germination index, seedling growth rate, and shoot length and reduced the proportion of abnormal seedlings. Whole-genome analysis placed RMT2NF4 within the genus *Priestia* and identified genes putatively associated with tryptophan biosynthesis, nutrient acquisition, motility, and stress adaptation. These genomic features suggest potential mechanistic associations with plant growth-promoting traits but do not confirm functional activity under the experimental conditions used in this study. Further biochemical assays, root colonization analysis, stress tolerance testing, and greenhouse or field validation are required to confirm the mechanisms underlying the observed seedling responses and to evaluate the practical bioinoculant potential of strain RMT2NF4.

## Figures and Tables

**Table 1:** Effects of *Priestia* sp. RMT2NF4 inoculation on seed quality and early seedling development.

Parameter	Control	RMT2NF4	Percentage Increasing	*p*-Value
Shoot length (cm)	12.63 ± 1.84 *	16.16 ± 4.83 *	+27.94%	0.0000
Root length (cm)	10.38 ± 2.00	12.38 ± 2.36	+19.33%	0.1688
Germination (%)	73.90 ± 11.72 *	83.70 ± 2.36 *	+13.26%	0.0026
GI (plant day^−1^)	11.29 ± 3.15 *	13.70 ± 2.03 *	+21.30%	0.0315
SGR (mg plant^−1^)	41.16 ± 3.56 *	50.95 ± 2.40 *	+23.77%	0.0461
Abnormal seedling (%)	21.8 ± 8.58	11.6 ± 7.21	−46.79%	0.2282

Data are presented as mean ± SD. *p*-values were obtained using one-way ANOVA comparing the uninoculated control and RMT2NF4-treated seeds. GI, germination index; SGR, seedling growth rate. Positive values indicate a percentage increase relative to the control, whereas negative values indicate a percentage decrease. * indicates statistically significant differences (*p* < 0.05). Values with *p* < 0.05 were considered statistically significant.

**Table 2:** General characteristics of the genome and annotation details of *Priestia* sp. RMT2NF4.

Feature	Value
Genome size (bp)	5,318,699
GC content (%)	37.89
Sequencing coverage	30.0×
Number of contigs	40
L50	3
N50 (bp)	811,292
Completeness (%)	99.27%
Contamination (%)	2.96%
Plasmids	0
Coding sequences (CDSs)	5698
rRNA genes	4
tRNA genes	68

## Data Availability

The data presented in this study are openly available in NCBI GenBank at https://www.ncbi.nlm.nih.gov/bioproject/?term=PRJNA1219766 (accessed on 5 February 2025), reference number [PRJNA1219766].

## Supplementary Materials

The following supporting information can be downloaded at: https://www.mdpi.com/article/10.3390/microorganisms14071388/s1, Supplementary Table S1: Plant growth-promoting (PGP) traits and associated genes identified from the whole-genome analysis of strain RMT2NF4. Supplementary Table S2. Accession numbers of genomes used for comparative analysis. Supplementary Table S3. Experimental design for the seed germination and seedling quality assays.
