# Supplementary material for: Enhancement of Sweet Corn Seed Quality and Early Seedling Vigor by Priestia sp. RMT2NF4: Functional and Genomic Characterization of a Plant Growth-Promoting Strain
Source: Microorganisms. 2026 Jun 23;14(7):1388. doi: 10.3390/microorganisms14071388 (PMC13413799; doi:10.3390/microorganisms14071388)
Supplement: Supplementary file 1 [file microorganisms-14-01388-s001.zip › Supplementary Table S1.pdf]

Supplementary Table S1. Plant Growth Promoting Traits from the Whole Genome

PGP	WP.	Name gene	Gene product	Size
**IAA**	WP_047749878.1	*trpA*	tryptophan synthase subunit alpha	816
	WP_028411500.1	*trpB*	tryptophan synthase subunit beta	1215
	WP_176343447.1	*trpC*	indole-3-glycerol phosphate synthase trpC	768
	WP_028411502.1	*trpD*	anthranilate phosphoribosyltransferase	1026
	WP_028411503.1	*trpE*	anthranilate synthase component I	1515
	WP_028412367.1	*trpS*	tryptophan--tRNA ligase	990
	WP_028414821.1	nd	AEC family transporter	957
**tRNA isopentenyltransferase-related genes**				
	WP_034265185.1	*miaA*	tRNA (adenosine(37)-N6)-dimethylallyltransferase MiaA	951
	WP_416225818.1	*miaB*	tRNA (N6-isopentenyl adenosine(37)-C2)-methylthiotransferase MiaB	1530
**Nitrogen metabolism-related genes**	WP_013059625.1	nd	NifU family protein	231
	WP_033579632.1	*FixB*	electron transfer flavoprotein subunit alpha FixB family protein	981
	WP_016765685.1	*FixA*	electron transfer flavoprotein subunit beta FixA family protein	774
	WP_154973942.1	*fixH*	FixH family protein	399

PGP	WP.	Name gene	Gene product	Size
	WP_221832550.1	*fixH*	FixH family protein	465
	WP_394553974.1	*fixH*	FixH family protein	381
	WP_154973942.1	*fixH*	FixH family protein	394
**P**	WP_033579664.1	*pstS*	phosphate ABC transporter substrate-binding protein PstS family protein	885
	WP_013059300.1	*pstC*	phosphate ABC transporter permease subunit PstC	888
	WP_025750576.1	*pstA*	phosphate ABC transporter permease PstA	930
	WP_416225848.1	*pstB*	phosphate ABC transporter ATP-binding protein PstB	798
	WP_013059193.1	*phoU*	phosphate signaling complex protein PhoU	660
	WP_013056072.1	nd	PhoH family protein	1329
	WP_033579680.1	nd	PhoH family protein	975
	WP_028410795.1	*plsX*	phosphate acyltransferase PlsX	1005
	WP_176343449.1	*hisC*	histidinol-phosphate transaminase	1104
**Root colonization**				
**Chemotaxis**	WP_014458457.1	*cheW*	chemotaxis protein CheW	429
	WP_416226273.1	nd	methyl-accepting chemotaxis protein	2019
	WP_048020021.1	nd	chemotaxis protein	906
	WP_186819738.1	nd	methyl-accepting chemotaxis	1245
	WP_176343466.1	*cheW*	chemotaxis protein CheW	1980

PGP	WP.	Name gene	Gene product	Size
	WP_028411411.1	*cheD*	chemotaxis protein CheD	495
	WP_053001469.1	nd	methyl-accepting chemotaxis protein	1512
	WP_416225413.1	nd	methyl-accepting chemotaxis protein	1983
**Motility**	WP_176343464.1	*flhF*	flagellar biosynthesis protein FlhF	1098
	WP_033579790.1	*flhA*	flagellar biosynthesis protein FlhA	2037
	WP_176343463.1	*flhB*	flagellar biosynthesis protein FlhB	1083
	WP_047749904.1	*fliR*	flagellar biosynthetic protein FliR	777
	WP_033579787.1	*fliQ*	flagellar biosynthesis protein FliQ	270
	WP_028411420.1	*filP*	flagellar type III secretion system pore protein FliP	669
	WP_028411421.1	*filP*	flagellar biosynthetic protein FliO	642
	WP_255219746.1	*filiY*	flagellar motor switch phosphatase FliY	1131
	WP_034265235.1	*fliM*	flagellar motor switch protein FliM	1002
	WP_028411424.1	*fliL*	flagellar basal body-associated protein FliL	432
	WP_025750317.1	*flbd*	flagellar FlbD family protein	210
	WP_416225822.1	*fliK*	flagellar hook-length control protein FliK	2619
	WP_028411429.1	*fliJ*	flagellar export protein FliJ	444
	WP_165635188.1	*fliI*	flagellar protein export ATPase FliI	1314
	WP_028411431.1	*fliH*	flagellar assembly protein FliH	789
	WP_025750326.1	*fliG*	flagellar motor switch protein FliG	1020

PGP	WP.	Name gene	Gene product	Size
	WP_048019845.1	*fliF*	flagellar basal-body MS-ring/collar protein FliF	1593
	WP_028411433.1	*fliE*	flagellar hook-basal body complex protein FliE	291
**Flagellar assembly and motility-associated genes**	WP_033578789.1	*motA*	flagellar motor stator protein MotA	795
	WP_025752068.1	*motB*	flagellar motor protein MotB	834
	WP_028411434.1	*flgC*	flagellar basal body rod protein FlgC	450
	WP_028411435.1	*flgB*	flagellar basal body rod protein FlgB	390
	WP_048019847.1	*flgD*	flagellar hook assembly protein FlgD	429
	WP_014458441.1	*flgG*	flagellar basal body rod protein FlgG	825
	WP_416226127.1	*flgK*	flagellar hook-associated protein FlgK	1524
	WP_033580408.1	*flgL*	flagellar hook-associated protein FlgL	882
	WP_014457790.1	*flgM*	flagellar biosynthesis anti-sigma factor FlgM	255
	WP_223546706.1	*flgN*	flagellar protein FlgN	462
**Cold-shock protein**	WP_013056405.1	nd	cold-shock protein	204
	WP_047750334.1	nd	cold-shock protein	213
	WP_033580115.1	nd	cold-shock protein	201
	WP_013058502.1	nd	cold-shock protein	201
	WP_013055689.1	nd	cold-shock protein	198

PGP	WP.	Name gene	Gene product	Size
	WP_013056130.1	nd	cold-shock protein	204
	WP_013056175.1	*cspD*	cold-shock protein CspD	204
	WP_013056409.1	nd	cold-shock protein	201
**Heat shock proteins**	WP_416226136.1	*smpB*	SsrA-binding protein SmpB	468
**Drought resistance**	WP_416225729.1	*chaB*	ChaB family protein	198
	WP_028413398.1	*proB*	glutamate 5-kinase	1107
	WP_013085533.1	*proB*	glutamate 5-kinase	825
	WP_176521382.1	*betB*	betaine-aldehyde dehydrogenase	1491
	WP_014458669.1	*trkH*	TrkH family potassium uptake protein	1320
	WP_255285406.1	*trkH*	TrkH family potassium uptake protein	1380
	WP_034270151.1	*kdpDN*	KdpD-like non-kinase potassium sensor	1149
	WP_034270154.1	*kdpC*	potassium-transporting ATPase subunit KdpC	567
	WP_416225542.1	*kdpB*	potassium-transporting ATPase subunit KdpB	2076
	WP_416225691.1	*kdpA*	potassium-transporting ATPase subunit KdpA	1662
	WP_080754503.1	*kdpF*	K(+)-transporting ATPase subunit F	87
