# Supplementary material for: Enhancement of Sweet Corn Seed Quality and Early Seedling Vigor by Priestia sp. RMT2NF4: Functional and Genomic Characterization of a Plant Growth-Promoting Strain
Source: Microorganisms. 2026 Jun 23;14(7):1388. doi: 10.3390/microorganisms14071388 (PMC13413799; doi:10.3390/microorganisms14071388)
Supplement: Supplementary file 1 [file microorganisms-14-01388-s001.zip › Supplementary Table S2.pdf]

Supplementary Table S2. Accession numbers of genomes used for comparative analysis.

**No.**	**Isolate**	**Accession**
**1**	*Bacillus megaterium* NBRC 15308 = ATCC 14581	PRJNA605350
**2**	*Bacillus megaterium* DSM 319	PRJNA42425
**3**	*Bacillus megaterium* QM B1551	PRJNA30165
**4**	*Bacillus flexus* NBRC 15715	PRJDB1313
**5**	*Bacillus* sp. SGD-V-76	PRJNA302099
**6**	*Bacillus nealsonii* AAU1	PRJNA168247
**7**	*Bacillus* sp. G2(2012b)	PRJEB623
**8**	*Domibacillus enclensis* strain NIO-1016	PRJNA373956
**9**	*Listeriaceae bacterium* FSL S10-1204	PRJNA182529
**10**	*Bacillus* sp. FF3	PRJEB4275
**11**	*Bacillus atrophaeus* HAB-5	PRJNA1004908
**12**	*Priestia aryabhattai* KNU45	PRJNA1304254
**13**	*Priestia* sp. TSO9	PRJNA772765
**14**	*Bacillus megaterium* STB1	PRJNA428341
