# Supplementary material for: Enhancement of Sweet Corn Seed Quality and Early Seedling Vigor by Priestia sp. RMT2NF4: Functional and Genomic Characterization of a Plant Growth-Promoting Strain
Source: Microorganisms. 2026 Jun 23;14(7):1388. doi: 10.3390/microorganisms14071388 (PMC13413799; doi:10.3390/microorganisms14071388)
Supplement: Supplementary file 1 [file microorganisms-14-01388-s001.zip › Supplementary Table S3.pdf]

Supplementary Table S3. Experimental design for the seed germination and seedling quality assays

Component	Description
**Crop**	Sweet corn (*Zea mays* L. saccharata), commercial hybrid cultivar 'Hi-Brix 59'
**Treatments**	Control: sterile distilled water; RMT2NF4: bacterial suspension
**Bacterial strain**	*Priestia* sp. RMT2NF4
**Cell density**	Approximately 1×10^{7} CFU mL^{-1}
**Seed treatment**	Seed soaking for 30 min
**Germination method**	Between-paper method following ISTA guidelines
**Replication**	20 independent replicates per treatment, 50 seeds per replicate
**Total seeds**	1,000 seeds per treatment; 2,000 seeds in total
**Incubation condition**	25 °C for 7 days
**Measurements**	GI on days 4 and 7; GP, SGR, shoot length, root length, abnormal seedlings on day 7
